# Real-time behavioral monitoring of C57BL/6J mice during reproductive cycle

**DOI:** 10.3389/fnins.2025.1509822

**Published:** 2025-03-03

**Authors:** Ariane Khatiz, Cassidy Tomlinson, Bohdana Ruzhytska, Erika Kathe Croft, Abdelaziz Amrani, Shannon Dunn, Adrianna Mendrek, Denis Gris

**Affiliations:** ^1^Program of Physiology, University of Sherbrooke, Sherbrooke, QC, Canada; ^2^Program of Immunology, University of Sherbrooke, Sherbrooke, QC, Canada; ^3^Program of Translational Medical Bioengineering, National Technical University of Ukraine, Kyiv, Ukraine; ^4^Department of Pediatrics, University of Sherbrooke, Sherbrooke, QC, Canada; ^5^Department of Immunology, University of Sherbrooke, Sherbrooke, QC, Canada; ^6^Department of Immunology, University of Toronto, Toronto, ON, Canada; ^7^Biological Platform, Sunnybrook Research Institute, Toronto, ON, Canada; ^8^Department of Psychology, Bishop’s University, Sherbrooke, QC, Canada; ^9^Department of Physiology and Pharmacology, University of Sherbrooke, Sherbrooke, QC, Canada

**Keywords:** behavior, sex, estrous, mouse model, male, female

## Abstract

**Introduction:**

The present study aims to identify differences in behavioral profiles in post-pubertal C57BL/6J males and female mice across distinct phases of the reproductive cycle in a home cage environment.

**Methods:**

To reduce human bias, we used an automated behavioral analysis system *HomeCageScan* from CleverSys Inc. Mice were monitored continuously, and resulting data were summarized across 24-h, light, and dark cycles. Behavioral activities of each period were analyzed using hierarchical clustering, factor analysis, and principal component analysis.

**Results:**

Females exhibited higher levels of physically demanding activities, including ambulatory and exploratory movements, particularly during estrus and metestrus, with estrus showing up to 30% more activity than males. In contrast, males consistently engaged in more sleep-related behaviors across all phases, with significantly higher engagement during the light cycle compared to females in proestrus and estrus (*p* < 0.0001); the extent of this sex difference was greater during proestrus and estrus than in metestrus and diestrus (*p* < 0.01). Notably, distinct patterns of sleep fragmentation were observed, with females experiencing greater disruptions during the light cycle, while males showed similar disruptions during the dark cycle. Feeding and resourcing behaviors were highest in males, showing up to 20% increase compared to cycling females, as well as significantly engaging in habituation-related behaviors such as feeding and digging. Interphase differences were observed within females, such as a significant increase of habituation-related activities during estrus compared to proestrus and diestrus (*p* < 0.05), while during the dark cycle, these activities peaked during the diestrus phase (*p* < 0.05). Female mice in the metestrus phase exhibited more sleep-related behaviors than those in proestrus.

**Discussion:**

Our study has revealed prevalent behavioral differences due to sex, and inter-phase variations by employing a continuous monitoring approach designed to reduce bias. This methodology ensures a comprehensive understanding of natural behavioral patterns and strategies.

## Introduction

Despite significant policy efforts, such as the NIH (1993) and the NIH’s mandate on considering sex as a biological variable (2015), the underrepresentation of females in biomedical research persists (Zucker and Beery, 2022; National Institutes of Health, 2015; Institute of Medicine (US) Committee on Ethical and Legal Issues Relating to the Inclusion of Women in Clinical Studies, 1994). While female participation in clinical trials has increased, recent analyses indicate that preclinical studies continue to exhibit a male bias, particularly in fields like neuroscience, pharmacology, and physiology (Woitowich et al., 2020; Zucker and Beery, 2022). For instance, a survey of publications in 2019 revealed that although the inclusion of both sexes has improved since 2009, male bias remains prevalent, and less than half of studies analyze results with sex as a factor (Woitowich et al., 2020). A persistent assumption contributing to this bias is that hormonal fluctuations in females introduce significant variability in experimental studies, a notion that has been challenged and discredited (Beery, 2018; Prendergast et al., 2014). The present study aims to address this assumption by comparing the behavioral profiles of male and female mice across various phases of the reproductive cycle.

Mice were chosen as the animal model for this research due to their widespread use in laboratory studies (Hickman et al., 2017). Female mice display hormonal fluctuations and reproductive cycling similar to humans, albeit their reproductive cycles are shorter (Caligioni, 2009). Female mice reach sexual maturity at 6–8 weeks and their estrous cycle in female mice lasts between 4 and 6 days (Weber and Olsson, 2008). Similar to the human cycle, the murine reproductive cycle is divided into the follicular and luteal phases, which involve egg maturation, release, and uterine lining thickening (Kim et al., 2009). These phases are further divided into four stages: (1) proestrus, (2) estrus, (3) metestrus, and (4) diestrus. While the four stages of the murine estrous cycle are defined by the fluctuating ovarian steroids and hormones, the cell types present in the vaginal canal reflect the endocrine activities and are how the groups are defined in this paper (Cora et al., 2015; McLean et al., 2012).Contemporary ethology often relies on predefined behavioral tests to assess cognitive or physical functions in mice, such as the Open Field test, Social Interaction test, Elevated Maze, and Rotarod test. These tests query different behavioral and cognitive domains. The Open Field test evaluates locomotion, hyperactivity, as well as anxiety-like behaviors based on a mouse’s aversion to open areas (Kraeuter et al., 2019). However, factors like cage position (top vs. bottom shelf), ambient temperature, lighting, and scent traces can influence test outcomes (Izídio et al., 2005; Saré et al., 2021). Rigorous standardization is critical to avoid bias from variables like experimenter factors such as experience and personal cues, test duration, and time of day. Variability in animal strain, age, estrous cycle, and environment also complicate reproducibility in studies in female rodent behavior studies. Despite inconsistencies across labs, it has become clear that estradiol levels are a significant factor impacting outcomes in assessment of spatial learning and memory in rodents (Sandstrom and Williams, 2001).

A study by Meziane and colleagues on the relationship between the estrous cycle and behavior in mice revealed notable variations in performance in BALB/cBYJ females across different estrous phases in several tests, including open field, tail flick, and tail suspension (2007). Specifically, these mice displayed increased exploratory behavior during the proestrus phase, suggesting lower anxiety levels. In contrast, C57BL/6 J females, preferred for their genetic modifiability, showed similar behaviors across estrous phases, except for tail suspension performance. However, these tests can introduce biases due to new, stressful situations and handling environments. To reduce these biases, our study employed a continuous behavioral evaluation tool that monitors mice within their home cage environment. This approach minimizes stress associated with handling and exposure to novel settings, which are known to affect behavioral outcomes (Guilloux et al., 2011; Tecott and Nestler, 2004). Although some environmental factors like cage positioning can still introduce variability, home cage monitoring significantly enhances the ecological validity of behavioral assessments by allowing observations in a familiar setting (Richter, 2020).

Yamamoto et al. (2018) studied the mouse ethome and investigated home cage behavior using automated behavioral assessment software. The study provided a robust framework for classifying mouse behavior into meaningful categories using dimensionality reduction approaches. Their analysis categorized behaviors into five clusters—sleep-related, exploratory-like, physically demanding, habituation-like, and nourishment—and six factors based on parallel analysis. This classification system considered the complexity and interrelated nature of mouse behaviors, offering a reproducible method to categorize activities. Building on this foundational work, we aimed to explore behavioral differences across sex and within the estrous cycle in female mice. By focusing on home cage monitoring, Yamamoto’s work highlighted the advantages of naturalistic behavior recording, which allows for the unbiased capture of spontaneous and ecologically valid behaviors while reducing the stress and variability introduced by traditional test-based assessments.

Our study expands upon this approach, utilizing HomeCageScan software to automate continuous monitoring of mouse behaviors, enabling comprehensive 24-h data collection and analysis. The system is designed to minimize the human factor, reduce the potential for observer bias, and increase the accuracy and reliability of the collected behavioral data. HomeCageScan has been validated for its ability to reliably detect and differentiate behavioral changes in rodent models, where it produced comparable results to conventional manual analysis in evaluating pain and analgesic effects in mice (Roughan et al., 2009). Previous studies have demonstrated its utility in evaluating behavioral phenotypes and treatment effects in rodent models (Steele et al., 2007; Dickinson et al., 2009; Roughan et al., 2009). The system allows for rapid and prolonged behavioral assessments, which can help identify less frequent behaviors and their temporal dynamics. By doing so, we provide a detailed investigation of behavioral patterns across the sexes and reproductive phases, to further the field’s understanding of complex behavioral dynamics.

## Materials and methods

### Ethics

All experiments and procedures were conducted and approved by the Animal Care Committee of the Faculty of Medicine and Health Sciences (FMSS) at the Université de Sherbrooke, under the protocol 2022–3508.

### Mouse model

In this study, we utilized a total of 20 C57BL/6J mice (Charles River), comprising 11 females and 9 males, all aged 8–9 weeks at the start of the experiment. Mice were housed individually in standard home cages under controlled environmental conditions. The facility maintains a 14:10 (lights on from 6:00 am to 8:00 pm) cycle as part of its standard operating procedures.

### Behavioral assessment tool

Behavioral data were collected continuously using Swann Pro Series HD720p cameras positioned in front of the cages. The female mice were recorded for a total of 110 days, with individual recording durations ranging from 9 to 10 days per mouse. The male mice were recorded for a total of 72 days. Due to acclimation effects on the first day of individual housing (Gris et al., 2017), we excluded data from the first day of recording to account for potential irregularities.

Data analysis was performed using an automated behavioral assessment tool, CleverSys Inc.—HomeCageScan, automatically analyzing the videos in 24-h periods on a 30 Hz resolution. The software detects and categorizes up to 35 unique activities, as shown in Supplementary Table 1, along with each activity’s respective definitions. Without human intervention, it detects various activities, including locomotor activity, feeding, drinking, rearing, and other specific activities of interest. Prior studies have demonstrated the ability of HomeCageScan to detect and categorize a wide range of behaviors, including fine motor activities such as grooming and sniffing (Schaefer and Claridge-Chang, 2012; Bains et al., 2018).

### Data analysis

Data were summarized in terms of 24-h, light cycle and dark cycle summaries. The raw data was processed using Python with various packages such as Pandas, SciPy, Matplotlib, Statsmodel, and Sklearn. Following Yamamoto and colleagues foundational work mentioned previously, hierarchical clustering and factor analysis was used to classify mouse behaviors into clusters and factors based on their statistical relationship (2018). Behaviors were grouped using complete linkage methods to identify dissimilarities, and factor analysis was performed using varimax rotation to uncover underlying dimensions of behavior. This approach allows for the reduction of behavioral complexity, as previously shown by Yamamoto et al. (2018). Principal component analysis was done to reduce the dimensionality of the dataset and eliminate correlations between the variables. Behavioral activities were not normally distributed. To define normality, Shapiro and D’Agostino test were performed. We used Spearman correlation for the hierarchical clustering analysis.

Statistical analysis is performed using One way ANOVA tests followed by Fisher’s least significance difference (LSD) for multiple comparisons with a *p* < 0.05 cutoff for determining statistical significance.

### Vaginal cytology

**Figure 1:**
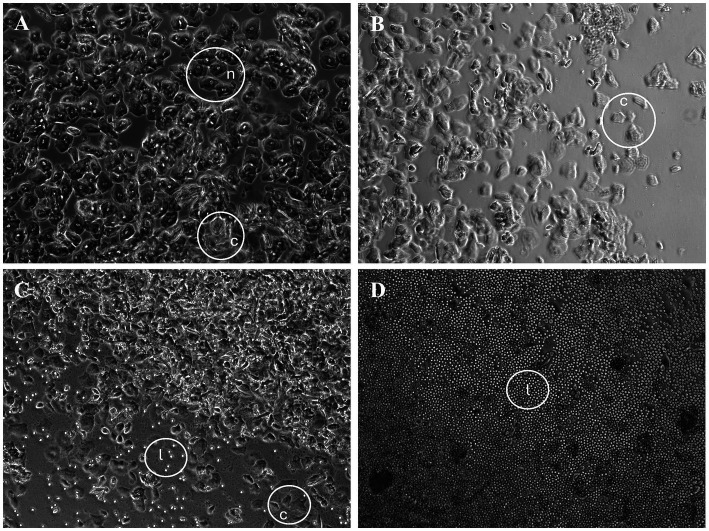
C57BL/6J vaginal cytology during the phases of the estrous cycle. **(A)** Proestrus with predominately round, nucleated epithelial cells (n). **(B)** Estrus with irregular-shaped, anucleated, cornified squamous epithelial cells (c). **(C)** Metestrus with anucleated, cornified epithelial cells and scattered neutrophils (l). **(D)** Diestrus with a dense population of neutrophils. Objective magnification is 20X.

Estrous was determined using vaginal lavage direct cytology on a wet mount and light microscopy as previously described (Byers et al., 2012). Vaginal smears were done at noon everyday (Figure 1). The proestrus phase is characterized by round, nucleated epithelial cells, which are often in clusters (Ajayi and Akhigbe, 2020; Cora et al., 2015; McLean et al., 2012). During the estrous phase, the cell population increases with predominately irregular-shaped, anucleated, cornified squamous epithelial cells with granular cytoplasm (Ajayi and Akhigbe, 2020; Cora et al., 2015). The epithelial cells are still found in clusters at the beginning but become larger and more dispersed near the end of the estrous phase (Ajayi and Akhigbe, 2020; Cora et al., 2015; McLean et al., 2012). The metestrus phase has combinations of neutrophils and large, non-granular, anucleated, keratinized epithelial cells (Ajayi and Akhigbe, 2020; Cora et al., 2015; McLean et al., 2012). Early in this phase, epithelial cells and neutrophils are about equal in ratio but later in the phase, cellularity will increase with darkly stained neutrophils outnumbering the epithelial cells 10 to 1 (Ajayi and Akhigbe, 2020; Cora et al., 2015; McLean et al., 2012). During the diestrus phase, overall cellularity decreases with the scattered population composed of mostly neutrophils, some nucleated epithelial cells, and low numbers of anucleated keratinized cells (Ajayi and Akhigbe, 2020; Cora et al., 2015). Near the end of this phase, the epithelial cells will become rounder and form small clusters in preparation for the proestrus phase (Cora et al., 2015). Vaginal cytology is a widely accepted and reliable method to determine the stages of the murine estrus cycle that is inexpensive and non-invasive.

The observed occurrences for each phase were as follows: Proestrus (P) = 21, Estrus (E) = 27, Metestrus (M) = 37, and Diestrus (D) = 25. To control for potential handling-induced behavioral changes, male mice were also handled similarly at the same time each day. The estrous stage identified from the vaginal smear collected at noon was considered to represent the estrous stage for the following 24-h period, including both the light and subsequent dark cycles. Therefore, behavioral data collected from noon on the day of the lavage until the next lavage at noon the following day were associated with the estrous stage determined at the time of the lavage.

## Results

### Hierarchical clustering and dendrogram

Using the dendrogram, the clusters are grouped in hierarchical order, becoming increasingly dissimilar. We used the Spearman correlation matrix-based hierarchical clustering as an exploratory tool for data analysis in three ways: 24-h bins, light cycle analysis, and dark cycle analysis.

### Hierarchical clustering analysis—24-h bins

**Figure 2:**
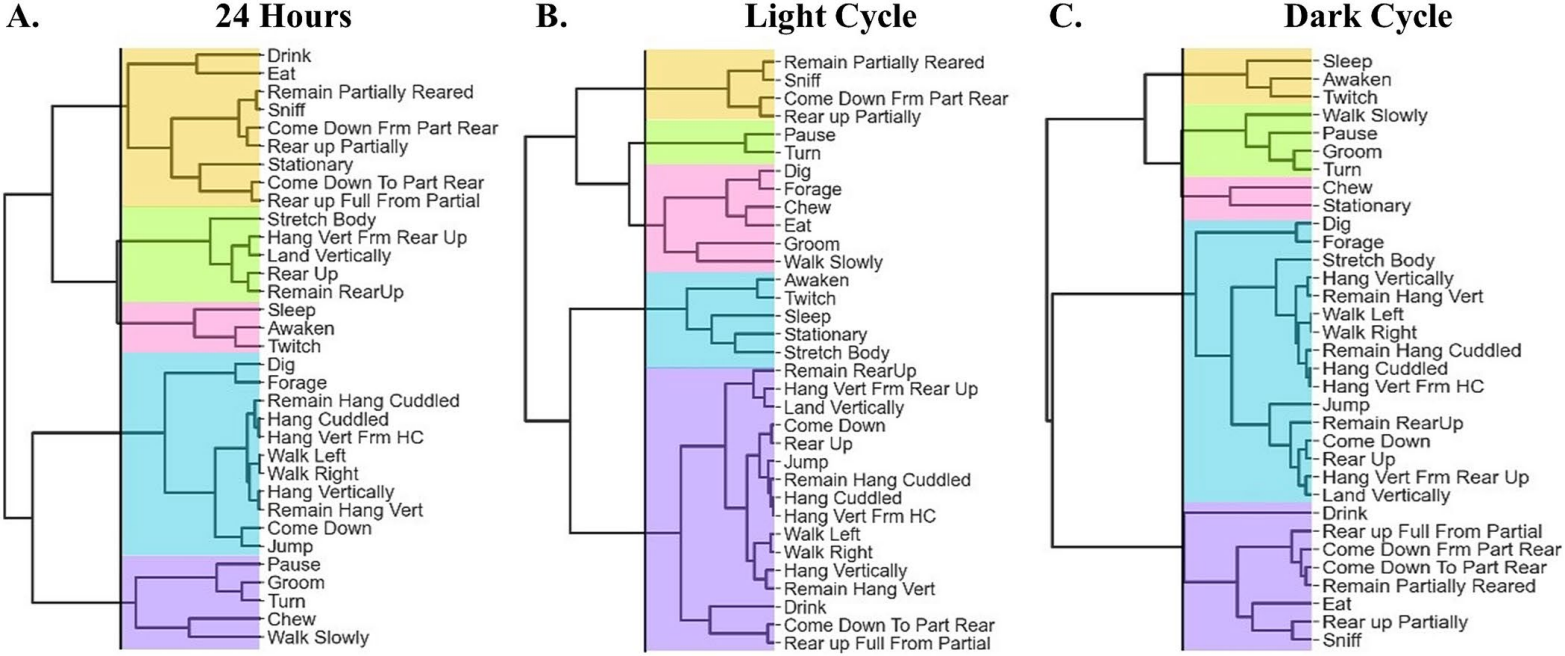
The hierarchical clustering correlation matrix between behavioral activities and the dendrogram in a 24-h dataset. **(A)** Cluster 1 (orange): Feeding and Resource Interaction. Cluster 2: Exploratory (green). Cluster 3 (pink): Sleep-Related Cluster. Cluster 4 (blue): Physically Demanding Cluster. Cluster 5 (purple): Habituation-Like Activities. **(B)** Light cycle clustering analysis. Cluster 1 (orange): Habituation Cluster, Cluster 2 (green): Idling Cluster, Cluster 3 (pink): Nourishment and Resource interaction Cluster, Cluster 4 (blue): Sleep-related Cluster, and Cluster 5 (purple): Physically Demanding. **(C)** Dark cycle clustering analysis Cluster 1 (orange): Sleep-Related, Cluster 2 (green): Habituation Cluster, Cluster 3 (pink): Idling Cluster, Cluster 4 (blue): Physically Demanding Cluster, and Cluster 5 (purple): Feeding and Resource Interaction.

**Figure 3:**
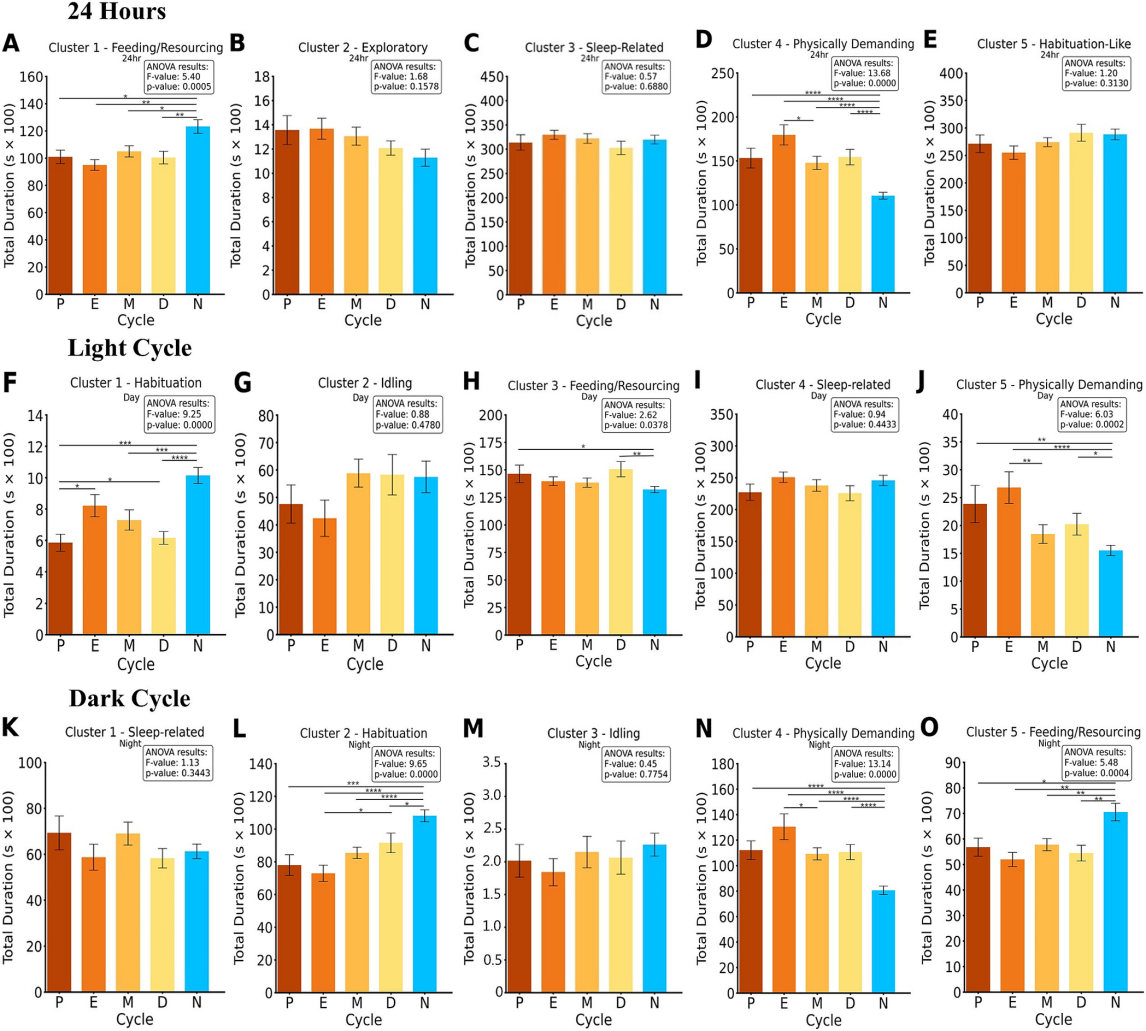
ANOVA and Fisher’s LSD *post-hoc* of clustered groups of activity summed into 24-h, light and dark cycle bins per cycle phase and control. The duration of each cluster is compared for each; proestrus (P), estrus (E), metestrus (M), diestrus (D), and the male control group (N). The results of the ANOVA and *p*-value are presented for each cluster. 24-h analysis clusters **(A–E)** revealed significant differences in Cluster 1 and 4. Light cycle analysis clusters **(F–J)** revealed significant differences in Cluster 1, 3, and 5. Dark cycle cluster analysis **(K–O)** revealed significant differences in Cluster 2, 4, and 5. *p < 0.05, **p < 0.01, ***p < 0.001, ****p < 0.0001.

For this analysis, we summarized the duration of all activities over a 24-h period. Using a hierarchical clustering correlation matrix and dendrograms to group the activities, five clusters were identified (Figure 2A). Cluster 1 (Figure 3A), termed the Feeding and Resource Interaction Cluster, comprises behaviors associated with feeding, drinking, and moderate activity, including Drink, Eat, Sniff, Come Down From Part Rear, Rear Up Partially, Stationary, Come Down To Part Rear, and Rear Up Full From Partial. Cluster 2 (Figure 3B), the Exploratory Cluster, includes activities that reflect physical effort and movement, such as Stretch Body, Hang Vertically From Rear Up, Land Vertically, Rear Up, and Remain Rear Up. Cluster 3 (Figure 3C), named the Sleep-Related Cluster, encompasses rest-oriented behaviors including Sleep, Awaken, and Twitch. Cluster 4 (Figure 3D), the Physically Demanding Cluster, reflects exploratory and resource-seeking behaviors, which include Dig, Forage, Remain Hang Cuddled, Hang Cuddled, Hang Vertically From Hang Cuddled, Walk Left, Walk Right, Hang Vertically, Remain Hang Vertically, Come Down, and Jump. Lastly, Cluster 5 (Figure 3E), the Habituation-Like Activities Cluster, focuses on self-maintenance and low-energy behaviors such as Pause, Groom, Turn, Chew, and Walk Slowly.

Statistical analysis of Cluster 1 revealed significant differences across groups, with an overall ANOVA result of *F* = 5.40, *p* < 0.001. Post-hoc Fisher’s LSD tests indicated that N exhibited significantly different behaviors compared to M (*p* < 0.05), D (*p* < 0.01), P (*p* < 0.05), and E (*p* < 0.01). These results suggest notable behavioral distinctions between males and females across reproductive phases in this cluster over the 24-h period. Cluster 4 revealed significant differences across groups, with an overall ANOVA result of *F* = 13.68, *p* < 0.001. *Post-hoc* Fisher’s LSD tests indicated that N exhibited significantly different behaviors compared to all female reproductive phases, including E (*p* < 0.001), M (*p* < 0.001), P (*p* < 0.001), and D (*p* < 0.001). Additionally, E differed significantly from M (*p* < 0.05).

### Hierarchical clustering analysis—light cycle

A light cycle analysis yielded five clusters derived from a correlation matrix and dendrogram (Figure 2B). Cluster 1 (Figure 3F), termed the Habituation Cluster, includes low-energy exploratory activities such as Remain Partially Reared, Sniff, Come Down From Part Rear, and Rear Up Partially. Cluster 2 (Figure 3G), the Idling Cluster, consists of idle activities such as Pause and Turn. Cluster 3 (Figure 3H), the Nourishment and Resource Interaction Cluster, encompasses feeding and resource-related activities including Dig, Forage, Chew, Eat, Groom, and Walk Slowly. Cluster 4 (Figure 3I), the Sleep-Related Cluster, features rest and recovery activities such as Awaken, Twitch, Sleep, Stationary, and Stretch Body. Finally, Cluster 5 (Figure 3J), the Physically Demanding Cluster, is characterized by high-energy and physically intensive activities, including Remain Rear Up, Hang Vertically From Rear Up, Land Vertically, Come Down, Rear Up, Jump, Remain Hang Cuddled, Hang Cuddled, Hang Vertically From Hang Cuddled, Walk Left, Walk Right, Hang Vertically, Remain Hang Vertically, Drink, Come Down To Part Rear, and Rear Up Full From Partial.

Statistical analysis of the light cycle revealed significant differences across groups in multiple clusters. For Cluster 1, an ANOVA result of *F* = 9.25, *p* < 0.001 indicated significant differences across groups. Post-hoc Fisher’s LSD tests showed that N differed significantly from M (*p* < 0.001), P (*p* < 0.001), and D (*p* < 0.001). Additionally, E differed significantly from P (*p* < 0.05) and D (*p* < 0.05). In Cluster 3, significant differences were observed with an ANOVA result of *F* = 2.62, *p* = 0.038. Post-hoc tests indicated that N differed significantly from D (*p* < 0.01) and P (*p* < 0.05). For Cluster 5, significant group differences were observed with an ANOVA result of *F* = 6.03, *p* < 0.001. *Post-hoc* analyses revealed that N differed significantly from E (*p* < 0.001), P (*p* < 0.01), and D (*p* < 0.05), while E also differed significantly from M (*p* < 0.01).

### Hierarchical clustering analysis—dark cycle

In the dark cycle analysis, five distinct behavioral clusters were identified (Figure 2C). Cluster 1 (Figure 3K), the Sleep-Related Cluster, includes low-energy behaviors associated with rest and minimal movement, such as Sleep, Awaken, and Twitch. Cluster 2 (Figure 3L), the Habituation Cluster, captures cautious exploratory and self-maintenance behaviors, including Walk Slowly, Pause, Groom, and Turn. Cluster 3 (Figure 3M), termed the Idling Cluster, is characterized by stationary and low-activity behaviors such as Chew and Stationary. Cluster 4 (Figure 3N), the Physically Demanding Cluster, includes physically intensive activities such as Dig, Forage, Stretch Body, Hang Vertically, Remain Hang Vertically, Walk Left, Walk Right, Remain Hang Cuddled, Hang Cuddled, Hang Vertically From Hang Cuddled, Jump, Remain Rear Up, Come Down, Rear Up, Hang Vertically From Rear Up, and Land Vertically. Lastly, Cluster 5 (Figure 3O), the Feeding and Resource Interaction Cluster, encompasses activities related to feeding and interacting with the environment, including Drink, Rear Up Fully From Partial, Come Down From Partially Reared, Come Down To Partially Reared, Remain Partially Reared, Eat, Rear Up Partially, and Sniff.

Statistical analysis of the dark cycle revealed significant differences across groups in multiple clusters. For Cluster 2, an ANOVA result of *F* = 9.65, *p* < 0.001 indicated significant differences across groups. *Post-hoc* Fisher’s LSD tests showed that N differed significantly from D (*p* < 0.05), P (*p* < 0.001), M (*p* < 0.001), and E (*p* < 0.001). Additionally, E differed significantly from D (*p* < 0.05). In Cluster 4, significant differences were observed with an ANOVA result of *F* = 13.14, *p* < 0.001. *Post-hoc* analyses revealed that N exhibited significantly different behaviors compared to E, M, P, and D (all *p* < 0.001), while E also differed significantly from M (*p* < 0.05). For Cluster 5, significant group differences were observed with an ANOVA result of *F* = 5.48, *p* < 0.001. Post-hoc analyses indicated that N exhibited significantly different behaviors compared to E, M, D (*p* < 0.01) and P (*p* < 0.05).

### Factor analysis

Factor analysis was used to extract patterns in the variations of behavioral activities related to the female estrous phases and the male group. We conducted a factor analysis on a 24-h period, light cycle and dark cycle datasets.

**Figure 4:**
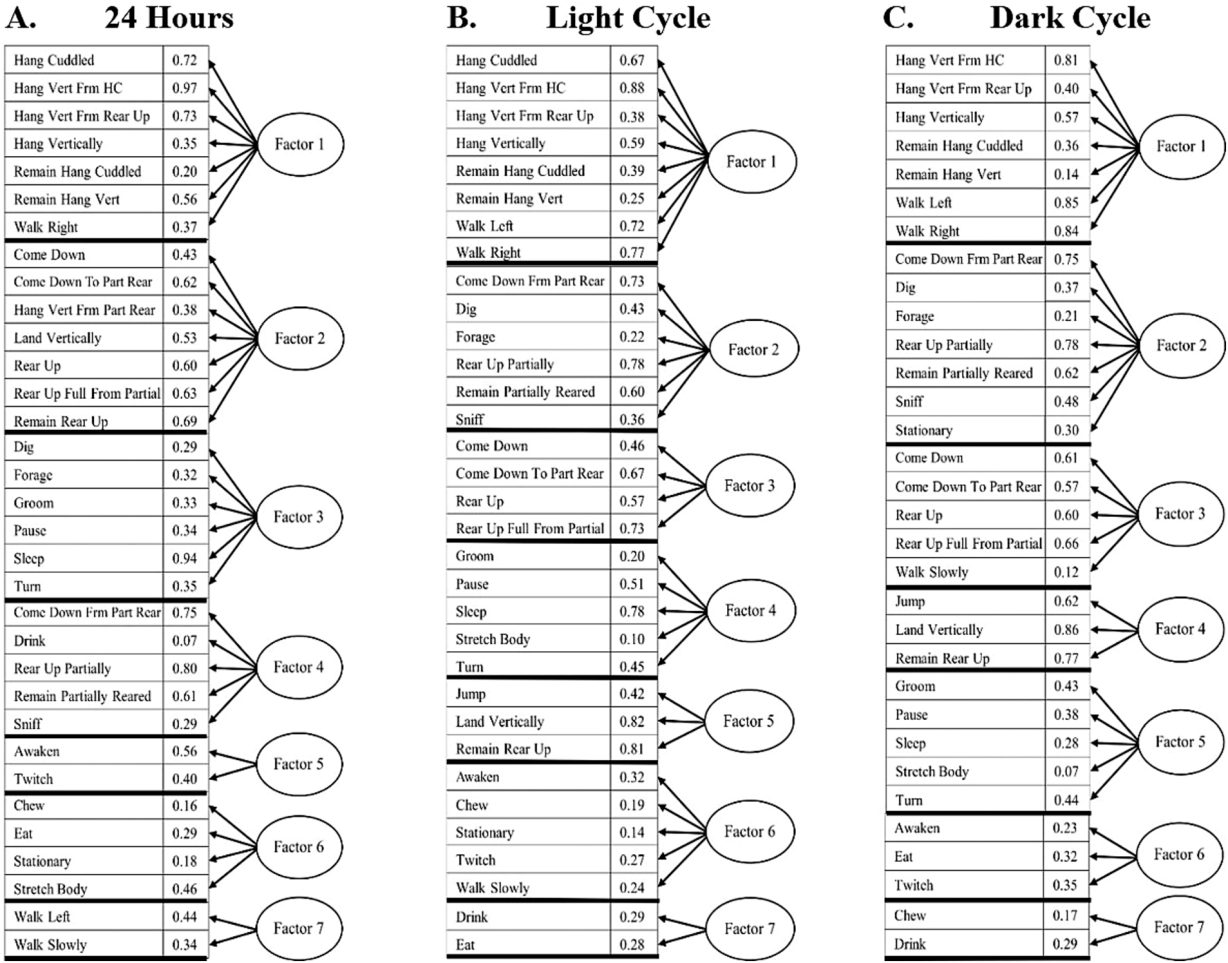
**(A)** 24-h, light cycle and dark cycle factor analysis, showing the activities included in each factor and their loadings. 24 Hours: Factor 1 – Exploratory, Factor 2 – Foraging, Factor 3 – Postural Locomotor, Factor 4 – Sleep-related, Factor 5 – Physically Demanding, Factor 6 – Pre/post Sleep, Factor 7 – Nourishment. **(B)** Light cycle: Factor 1 – Physically Demanding, Factor 2 – Postural Locomotor, Factor 3 – Sleep-Related, Factor 4 – Habituation, Factor 5 – Pre/Post Sleep, Factor 6 – Nourishment, Factor 7 – Exploratory. **(C)** Dark cycle: Factor 1 – Exploratory, Factor 2 – Foraging, Factor 3 – Postural Locomotor, Factor 4 – Physically Demanding, Factor – Sleep-related, Factor 6 – Pre/Post Sleep, Factor 7 – Nourishment.

**Figure 5:**
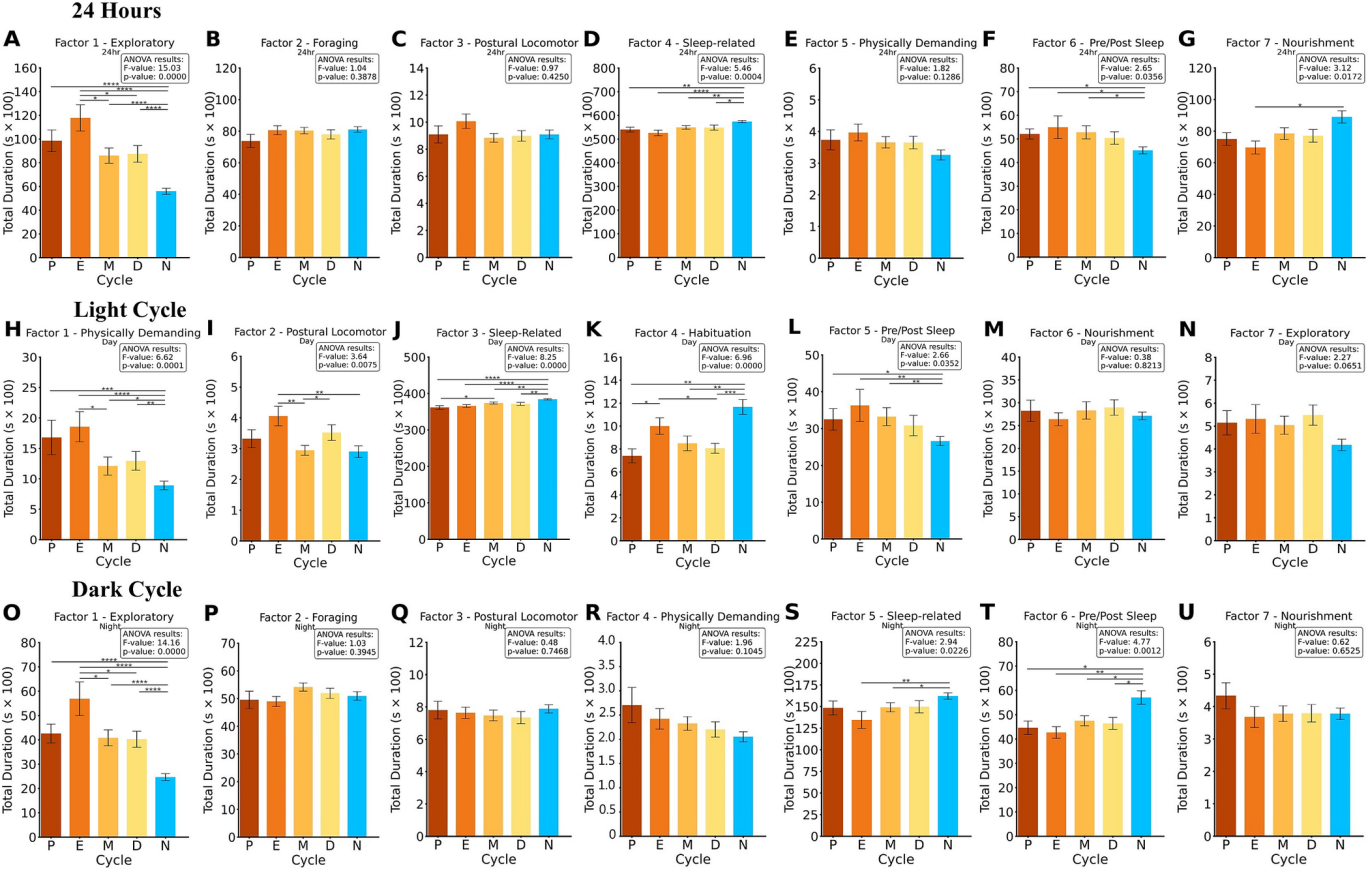
ANOVA and Fisher’s LSD *post-hoc* of factors summed into 24-h, light and dark cycle bins using Varimax rotation. The duration of the summed factors is compared for each, P, E, M D and N. The results of the ANOVA and *p*-value are presented for each cluster. 24-h factor analysis **(A–G)** revealed significant differences in Factor 1, 4, 6, and 7. In the light cycle factor analysis **(H–N)**, significant differences in Factor 1, 2, 3, 4, and 5. In the dark cycle factor analysis **(O–U)**, significant differences in Factor 1, 5, 6. *p < 0.05, **p < 0.01, ***p < 0.001, ****p < 0.0001.

In the factor analysis for 24-h bins, seven distinct behavioral factors were identified (Figure 4A). Factor 1 (Figure 5A) the Exploratory Factor, comprises behaviors associated with exploration and movement, including Hang Cuddled, Hang Vert From HC, Hang Vert From Rear Up, Hang Vertically, Remain Hang Cuddled, Remain Hang Vert, Walk Left, and Walk Right. Factor 2 (Figure 5B), the Foraging Factor, captures resource-seeking behaviors such as Come Down From Part Rear, Dig, Forage, Rear Up Partially, Remain Partially Reared, and Sniff. Factor 3 (Figure 5C), the Postural Locomotor Factor, reflects behaviors related to posture and transitions, including Come Down, Come Down To Part Rear, Rear Up, and Rear Up Full From Partial. Factor 4 (Figure 5D), the Sleep-Related Factor, includes rest and low-energy activities such as Groom, Pause, Sleep, Stretch Body, and Turn. Factor 5 (Figure 5E), the Physically Demanding Factor, is characterized by high-energy behaviors such as Jump, Land Vertically, and Remain Rear Up. Factor 6 (Figure 5F), the Pre/post Sleep Factor, focuses on behaviors that precede or follow resting behaviors, including Awaken, Chew, Stationary, Twitch, and Walk Slowly. Finally, Factor 7 (Figure 5G), the Nourishment Factor, includes behaviors directly related to feeding, such as Drink and Eat.

Statistical analysis of the 24-h factor data revealed significant differences across groups in multiple factors. For Factor 1, an ANOVA result of *F* = 15.03, *p* < 0.001 indicated strong group differences. Post-hoc Fisher’s LSD tests showed that N differed significantly from E, D, M, and P (all *p* < 0.001), while E also differed significantly from D (*p* < 0.05) and M (*p* < 0.05). For Factor 4, significant differences were observed with an ANOVA result of *F* = 5.46, *p* < 0.001. Post-hoc analyses revealed that N exhibited significantly different behaviors compared to E (*p* < 0.001), M (*p* < 0.01), D (*p* < 0.05), and P (*p* < 0.01). In Factor 6, an ANOVA result of *F* = 2.65, *p* < 0.05 indicated group differences. *Post-hoc* tests revealed that N differed significantly from M, E, and P (all *p* < 0.05). Finally, Factor 7 showed significant differences with an ANOVA result of *F* = 3.12, *p* < 0.05. *Post-hoc* analyses indicated that N differed significantly from E (*p* < 0.05). These results highlight distinct group differences across exploratory, sleep-related, habituation, and nourishment factors over the 24-h period.

In the light cycle-only analysis, seven distinct behavioral factors were identified, as shown in Figure 4B. Factor 1 (Figure 5H), the Physically Demanding Factor, includes high-energy activities such as Hang Cuddled, Hang Vert From HC, Hang Vertically, Jump, Remain Hang Cuddled, Remain Hang Vert, and Walk Right. Factor 2 (Figure 5I), the Postural Locomotor Factor, reflects behaviors related to posture transitions and vertical movement, including Come Down, Come Down To Part Rear, Hang Vert from Rear Up, Land Vertically, Rear Up, Rear Up Full From Partial, and Remain Rear Up. Factor 3 (Figure 5J), the Sleep-Related Factor, captures rest-oriented and low-energy activities, such as Dig, Forage, Groom, Pause, Sleep, and Turn. Factor 4 (Figure 5K), the Habituation Factor, comprises exploratory and maintenance behaviors like Come Down Frm Part Rear, Drink, Rear Up Partially, Remain Partially Reared, and Sniff. Factor 5 (Figure 5L), the Pre/Post Sleep Factor, focuses on behaviors associated with transitions into or out of rest, including Awaken and Twitch. Factor 6 (Figure 5M), the Nourishment Factor, includes feeding and stationary behaviors such as Chew, Eat, Stationary, and Stretch Body. Factor 7 (Figure 5N), the Exploratory Factor, highlights exploratory behaviors such as Walk Left and Walk Slowly.

Statistical analysis of the light cycle revealed significant differences across groups in multiple factors. For Factor 1, an ANOVA result of *F* = 6.62, *p* < 0.001 indicated significant group differences. *Post-hoc* Fisher’s LSD tests showed that N differed significantly from E (*p* < 0.001), P (*p* < 0.001), M (*p* < 0.05), and D (*p* < 0.01), while E also differed significantly from M (*p* < 0.05). For Factor 2, significant differences were observed with an ANOVA result of *F* = 3.64, *p* < 0.01. Post-hoc analyses revealed that N differed significantly from E (*p* < 0.01), and M differed from D (*p* < 0.05) and E (*p* < 0.01). In Factor 3, significant group differences were identified with an ANOVA result of *F* = 8.25, *p* < 0.001. Post-hoc tests indicated that N differed significantly from D (*p* < 0.01), P (*p* < 0.001), M (*p* < 0.01), and E (all *p* < 0.001), and M differed significantly from P (*p* < 0.05). For Factor 4, an ANOVA result of *F* = 6.96, p < 0.001 revealed significant differences. Post-hoc analyses showed that N differed significantly from P (*p* < 0.01), M (*p* < 0.01), and D (*p* < 0.001), while E also differed from P and D (both *p* < 0.05). Lastly, for Factor 5, significant group differences were observed with an ANOVA result of *F* = 2.66, *p* < 0.05. *Post-hoc* tests revealed that N differed significantly from P (*p* < 0.05), E, and M (both *p* < 0.01). These findings highlight group differences in exploratory, postural locomotor, sleep-related, and nourishment behaviors during the light cycle.

Similarly, a dark cycle factor analysis was conducted with seven distinct factors, as shown in Figure 4C. Factor 1 (Figure 5O), the Exploratory Factor, includes behaviors associated with exploration and movement, such as Hang Vert From HC, Hang Vert From Rear Up, Hang Vertically, Remain Hang Cuddled, Remain Hang Vert, Walk Left, and Walk Right. Factor 2 (Figure 5P), the Foraging Factor, captures resource-seeking and maintenance behaviors, including Come Down Frm Part Rear, Dig, Forage, Rear Up Partially, Remain Partially Reared, Sniff, and Stationary. Factor 3 (Figure 5Q), the Postural Locomotor Factor, reflects transitional and posture-related activities, such as Come Down, Come Down To Part Rear, Rear Up, Rear Up Full From Partial, and Walk Slowly. Factor 4 (Figure 5R), the Physically Demanding Factor, is characterized by high-energy behaviors, including Jump, Land Vertically, and Remain Rear Up. Factor 5 (Figure 5S), the Sleep-Related Factor, includes rest-oriented and low-energy activities, such as Groom, Pause, Sleep, Stretch Body, and Turn. Factor 6 (Figure 5T), the Pre/Post Sleep Factor, focuses on transitions into or out of rest states and includes Awaken, Eat, and Twitch. Factor 7 (Figure 5U), the Nourishment Factor, captures feeding-related behaviors, including Chew and Drink.

Statistical analysis of the dark cycle revealed significant differences across groups in several factors. For Factor 1, an ANOVA result of *F* = 14.16, *p* < 0.001 indicated strong group differences. Post-hoc Fisher’s LSD tests showed that N differed significantly from E, P, M, and D (all *p* < 0.001). Additionally, E differed significantly from M and D (both *p* < 0.05). For Factor 5, significant differences were observed with an ANOVA result of *F* = 2.94, p < 0.05. Post-hoc tests revealed that N differed significantly from M (*p* < 0.05) and E (*p* < 0.01). In Factor 6, significant group differences were identified with an ANOVA result of *F* = 4.77, *p* < 0.01. *Post-hoc* analyses indicated that N differed significantly from E (*p* < 0.01), P, M, and D (all *p* < 0.05). These findings highlight group differences in exploratory, habituation, and nourishment behaviors during the dark cycle.

### Principal component analysis (PCA)

**Figure 6:**
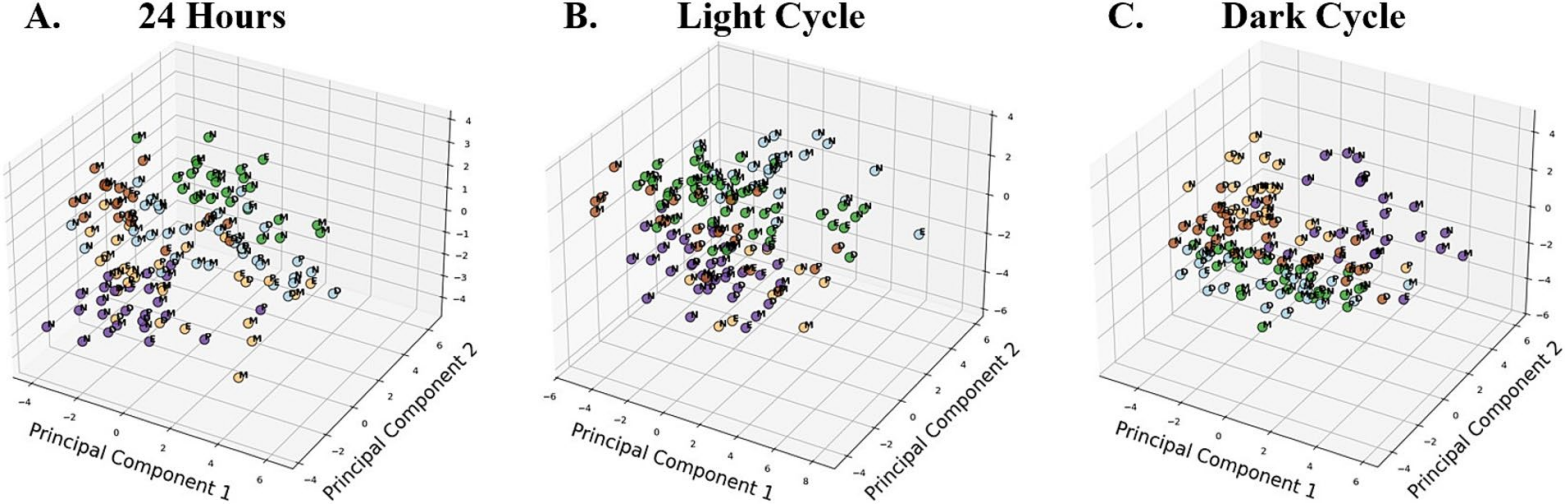
3D Scatter Plots of Principal Components 1, 2, and 3 illustrating K-means clustering (*k* = 5) across time periods, representing five behavioral groups. Data points are color-coded based on K-means cluster assignment, identifying groupings of similar activity patterns. Across all time periods, N was significantly different from all female phases in PC1 and PC2 (*p* < 0.05). The blue K-means clustering was identified as the dominant cluster for N in the 24-h **(A)** and light cycle **(B)** plots, while beige represented N in the dark cycle **(C)**. Additional differences were observed between E and M in the light cycle (*p* < 0.01). No significant cycle effect was found for PC3 and PC4.

Principal component analysis aims to extract the main orthogonal contributors called principal components to explain most of the variance of the data matrix analyzed (Cozzolino et al., 2019). This dimension-reduction technique summarizes the variables, which we conducted on a 24-h basis (Figure 6A), light cycle (Figure 6B) and dark cycle (Figure 6C), and standardized the dataset. K-Means clustering was applied to group the dataset into five clusters, corresponding to the five groups, to explore similarities in behavioral activities across different cycles.

An ANOVA was used to assess the impact of different cycles on the first four principal components (PC1, PC2, PC3, PC4) derived from the behavioral dataset. For PC1, the 24-h analysis revealed significant effects of the cycle for the total dataset (*p* < 0.001), light cycle (*p* = 0.002), and dark cycle (*p* = 0.008). *Post-hoc* Fisher’s LSD tests indicated that N differed significantly from all female reproductive phases (E, M, D, and P; *p* < 0.05) across all time periods. Additionally, during the light cycle, significant differences were observed between E and M (*p* = 0.004). For PC2, significant cycle effects were observed in the total dataset (*p* = 0.010), light cycle (*p* < 0.001), and dark cycle (*p* < 0.001). *Post-hoc* tests revealed that N differed significantly from all female phases (*p* < 0.05) across all time periods. No significant differences were observed among female phases themselves for PC2.

For PC3 and PC4, no significant cycle effects were found in any time period, suggesting that these components do not capture meaningful behavioral differences across cycles. These findings highlight the importance of PC1 and PC2 in distinguishing behavioral patterns across male and female phases, with particularly differences observed between males (N) and females (E, M, D, P) in all time periods.

### Activity across cycles and sexes

The activity analysis revealed significant differences in activity levels in females across different stages of the estrous cycle (P, E, M, D) and between the female and the male group. These differences provide insights into distinct behavioral patterns influenced by both sex and cycle phases. An ANOVA was conducted to compare the effects of different reproductive cycles on various activities, aggregated into 24-h bins, light and dark cycle analysis. Supplementary Table 2 showcases all the activity’s significant differences between the female cycles and the male group during the 24 h, light cycle and dark cycle analysis.

Significant differences were observed for certain inter-female cycles in specific activities when analyzing the activities across cycles. During the light cycle, the Come Down activity significantly differed between the M and E cycles (*p* < 0.01). The Hang Vertically from Rear Up was also distinct between the M and E cycles (*p* < 0.05). The Sniff activity showed variation between the D and E cycles (*p* < 0.01). Additionally, Walk Left and Walk Right differed significantly between the M and E cycles (*p* < 0.05). In the dark cycle analysis, hanging behaviors exhibited significant differences. Hang Cuddled varied between the M and E cycles (*p* < 0.01), and Remain Hang Cuddled differed between the M and E cycles (*p* < 0.05) as well as the D and E cycles (*p* < 0.05). Walking activities also showed distinctions between the M and E cycles (*p* < 0.05) and between the D and E cycles (*p* < 0.05).

## Discussion

The longstanding bias in preclinical trials favoring male models has notably limited our understanding of female health, particularly in behavioral research (Beery and Zucker, 2011; Lovick and Zangrossi, 2021). By exploring and comparing female and male mouse behavior when females were at different stages of the estrous cycle, we found that differences between male and female mouse behavior go beyond the stage of the estrous cycle. Most of the variability was associated with sex rather than the estrous cycle. The use of 24-h recording revealed that most of the differences between various groups of mice occur in the dark cycle.

### Advantages of continuous monitoring

The implementation of automated behavioral assessment software enabled continuous monitoring of mice over 9 days, providing an opportunity to address the controversies and inconsistencies in the literature regarding the effects of the estrous cycle on behavior. This approach also mitigates the lack of consensus on standardized procedures for studying female mice behavior. In this study, we used the C57BL/6J mouse strain, which is widely used due to its genetic modifiability and physiological similarities to humans. However, strain differences are important to consider. For instance, when compared to the BALB/c strain, C57BL/6J mice exhibit more stable performances across all four estrous phases in traditional behavioral tasks, including open field, rotarod, startle reflex, pre-pulse inhibition, tail flick, and hot plate tasks (Meziane et al., 2007).

CleverSys Inc. offers a comprehensive and unbiased method for analyzing behaviors in mice’s natural environment, contrasting with performance tasks that introduce artificial contexts and stimuli (Meziane et al., 2007). This tool reduces observer bias and enhances the accuracy and reliability of behavioral data.

The continuous monitoring provided by the software allowed us to detect subtle behavioral changes across the estrous phases, which may not have been observable using traditional methods. Furthermore, the sensitivity of this automated tool enables tracking health changes in mouse models, making it a valuable tool for studying diseases relevant to women’s health. The software also facilitates 24-h monitoring, capturing diurnal and nocturnal activity differences, which can vary by strain and sex (König et al., 2020).

### Benefits of analysis methods

To capture a comprehensive view of mouse behavior in a home cage environment, we performed analyses across three different time frames: 24-h, light cycle-only, and dark cycle-only. Each time frame offers unique insights into behavioral patterns that may remain undetected. The 24-h analysis provides a holistic view, allowing us to track behaviors across the entire light–dark cycle. This approach captures the mice’s diurnal and nocturnal activities, offering a more complete representation of their daily routines. The light cycle analysis focuses on behavior during the less active phase for nocturnal animals, revealing patterns driven by circadian rhythms and light exposure. In contrast, the dark cycle analysis highlights behaviors during the most active period for mice, offering detailed insights that are often subdued during the light cycle.

We employed three complementary methods to analyze the data comprehensively: hierarchical clustering, factor analysis, and PCA. Hierarchical clustering groups similar behaviors into clusters, allowing the categorization of activity patterns and revealing relationships between behavioral activities. On the other hand, factor analysis reduces the complexity of the dataset by identifying underlying factors that explain shared variance in behavior. This simplifies the large number of observed behaviors into key, interpretable factors. Additionally, PCA reduces the dimensionality of the data, retaining the most variance, transforming correlated variables into a set of uncorrelated principal component. This identifies the primary axes of behavioral differentiation between time frames and estrous phases, focusing on the most critical differences. These methods extracted more nuanced and subtle insights from the data, with overall 24-h trends and diurnal and nocturnal phases.

### Sex differences in behaviors

A key finding of this study is that males and females exhibit pronounced behavioral differences, suggesting that sex differences play a substantial role in shaping specific behavioral patterns. Previous studies have found that sex-based distinctions in behaviors and neural structures, such as in the locus coeruleus, played a significant role in shaping anxiety, pain sensitivity, and memory in mice (Mariscal et al., 2023). While hormonal fluctuations across the estrous cycle contribute to variability, our results highlight that sex-based distinctions are more prominent in this context.

Females exhibited higher levels of physically demanding activities compared to males (Figures 3D,J,N, 5H). This suggests that sex plays a critical role in driving increased physical activity in female mice, consistent with findings that female rodents’ activity levels can be 20–50% higher than males (Lightfoot, 2008). Additionally, it has been reported that females demonstrated 3 to 7 more counts of horizontal locomotion than males during the light and dark cycle across various mouse strains (König et al., 2020). It was also shown that regardless of gonadectomy, females exhibited more total locomotor activity and consumed more food than males at both feeding requirements, with extensive running activity beyond what was needed to generate food, especially among females (Perrigo and Bronson, 1985). This suggests that male and female house mice employ different strategies in relation to their behavior and reproductive development under existing foraging conditions, with females appearing more resource-dependent than males (Perrigo and Bronson, 1985). This may stem from sex-based differences in energy allocation, as male C57BL/6J mice show lower energy expenditure following weight loss and regain compared to females due to the activity of estradiol on the sex hormone receptor ERα in hypothalamic areas that regulate activity and metabolism (de Souza et al., 2022).

We also found that physically demanding activities were significantly higher in females during estrus when compared to metestrus and males. It is well established that the changing levels of estrogen through the estrous cycle influences many behaviors in mice, such as activity levels (Morgan and Pfaff, 2001; Morgan et al., 2004). These results are inconsistent with reports that state during the proestrus phase, where estrogen levels are at their peak, mouse physically demanding activities are also highest (Baker and Driver, 2007; Kopp et al., 2006; Morgan et al., 2004; Ogawa et al., 2003; Meziane et al., 2007). Other studies have found no difference in activity based on basal locomotion between estrous phases unlike our results determining a significant increase during the estrus phase (Zeng et al., 2023; Aguiar et al., 2018). Notably, these studies used tests involving short-term monitoring such as the open field test and mazes whereas present study utilizes long-term monitoring (Morgan et al., 2004; Aguiar et al., 2018; Morgan and Pfaff, 2001; Meziane et al., 2007). Estradiol (E2) drops significantly in tissues, skeletal muscle and serum as the mice transition from P to E, coinciding with ovulation and the potential for increased mate seeking behaviors which may explain the increased physical activity (Unger et al., 2023; Ajayi and Akhigbe, 2020; Alvord and Pendergast, 2024).

Males exhibited greater engagement in habituation activities during both the light and dark cycles. These habituation behaviors included drinking, digging, and grooming. Our findings revealed both sex-based differences, with males differing significantly from females in proestrus, metestrus, and diestrus, as well as inter-phase differences within females, such as higher habituation activity during estrus compared to proestrus and diestrus (Figures 3F, 3L, 5K). This pattern was consistent across both the light and dark cycles. Habituation refers to a reduced response to stimuli and diminished exploratory activity in a familiar environment (Tafreshiha et al., 2021). Previous research has also highlighted sex-specific tendencies in innate behaviors, noting that while burrowing and digging are inherent in both sexes, males demonstrate a stronger drive for digging than females (Pond et al., 2021). This suggests a sex-based preference in repetitive and familiar behaviors.

Male and female exhibit distinct sex-based differences in behavioral patterns more evident during the dark cycle, namely in exploratory activities. Our findings show that females across all phases consistently displayed higher levels of exploratory behavior than males. This suggests that females are more inclined to engage in environmental exploration, as observed in the 24 h and dark cycle, driven by sex-based differences, as well as significant inter-phase differences (Figures 5A,O). Previous research supports these findings, showing robust sex effects in ambulatory motor activity with grid-climbing behaviors, with females exhibiting higher activity levels than males, especially during the dark cycle (König et al., 2020; Borbélyová et al., 2019). While strain differences contributed to variability, the overall trend consistently favored females having higher activity levels, particularly during the dark cycle when sex differences were most pronounced (König et al., 2020; Leussis and Bolivar, 2006). Females maintain heightened curiosity or vigilance regardless of hormonal state. Moreover, prior research supports this conclusion, showing that female mice run approximately 20% farther per day than males across different strains (Lightfoot, 2008).

Males exhibit higher resource-seeking and feeding behaviors than females at all phases of the estrous cycle. From the factor analysis (Figure 5G), males exhibit higher nourishment behaviors, such as eating, drinking and rearing to reach these resources, than females in the estrus phase only. This is consistent with other reports of rodents tending to eat less during the estrus phase due to the sudden reduced levels of estrogen which may cause them to spend more time on mate-seeking behaviors (Alvord and Pendergast, 2024; Asarian and Geary, 2013). Cluster analysis showed that males engage in more feeding and resource interactions during the dark cycle than females at all phases (Figures 3A,O). This is consistent with mice being nocturnal animals that typically eat in the dark (Ellacott et al., 2010). In addition, it is consistent that males tend to consume as much food but spend more time doing so than females (Alvord and Pendergast, 2024; Maric et al., 2022). Notably, during the light cycle female mice in the proestrus and diestrus phases exhibit more feeding and resource interactions than males. This is partially consistent with the finding that the diestrus phase is the highest feeding phase for female mice (Asarian and Geary, 2013). The factor analysis for the individual light and dark cycles shows individual light and dark cycles’ factor analysis shows no significant difference in no significant difference for feeding behaviors. It is difficult to attribute feeding behaviors directly to the estrous cycle due to inconsistencies in reports of strain and even the influence of food preferences of mice, for example, female mice fed with honey-laced food ate more during estrus but less with chow (Asarian and Geary, 2013).

Females consistently exhibited higher postural locomotion activities than males throughout the light cycle. This difference was particularly pronounced during the estrus phase, as seen in Figure 5I, where females transitioned in posture more frequently than males. Our findings align with prior research demonstrating sex differences in spontaneous cage-related activities, such as grid climbing, in laboratory mice (Borbélyová et al., 2019). While these results suggest the sex-dependent nature of such behaviors, however, the exact functional significance of this heightened postural activity and grid-climbing remains to be elucidated. Moreover. this finding aligns with previous studies on C57BL/6 J mice strains, showing sex differences in activity levels (König et al., 2020; Rosenfeld, 2017). Research indicates that female mice remain more active than males even after gonadectomy, and estrogen supplementation restores activity levels in ovariectomized females to those seen in intact females, underscoring the role of estrogen in driving higher activity levels in females (Rosenfeld, 2017).

Female engaged in sleep-related behaviors significantly less than males across all phases. It was observed that females in estrus during all time periods, especially during the dark cycle, was significantly less than the males (Figures 5D,J,S). A similar trend was observed during the light cycle, though phase-specific differences emerged, with females in proestrus displaying distinct behavior compared to those in metestrus. This finding aligns with previous research demonstrating notable sex differences in the sleep–wake behavior and architecture of C57BL/6 J mice, where females exhibited less overall sleep, particularly during the dark period, with reduced NREM sleep, increased REM-like sleep, and more wakefulness compared to males (Mannino et al., 2024). Regarding pre- and post-sleep activities, our findings reveal that female mice were more engaged in these behaviors during the light cycle. In contrast, during the dark cycle, male mice exhibited greater involvement in pre- and post-sleep activities. This suggests that females are more sensitive of their environment during the light cycle, experiencing more pre/post sleep activities, causing them to rouse from sleep more often than male and having a fragmented sleep, which is consistent with previous research showing that females have shorter non-rapid eye movement sleep and increased wake time compared to males, reflecting differences in baseline sleep–wake behavior (Choi et al., 2021; Mannino et al., 2024).

### Estrous phases differences

Measuring exhaustive behavioral profiles allowed detection of behavioral differences associated with different estrous phases.

The analysis reveals significant inter-phase differences in high-energy and physically demanding activities, particularly between estrus and metestrus phases. Mice demonstrated a clear preference for engaging in more physically demanding activities during the estrous phase compared to the metestrus phase, and this pattern was consistent across different time frames, including 24-h observations, light cycle, and dark cycle (Figures 3D,J,N, 5H). This suggests estrus phases appear to drive higher energy expenditure during physically demanding activities. Previous studies have similarly reported sex differences in ambulatory motor activity and rearing behavior, with females generally exhibiting higher activity than males, particularly during the dark cycle (König et al., 2020). Although strain differences contribute to variability, the overall trend favors higher activity in females during high hormone phases, as shown by sex hormone effects in rodents (Lightfoot, 2008).

Exploratory behaviors varied across the estrous cycle, with distinct patterns observed during both 24-h and dark cycle periods. Females in estrus consistently exhibited higher exploratory activity compared to those in metestrus and diestrus (Figures 5A,O). This heightened activity may be attributed to estrous stage-related factors, as estrus is typically associated with elevated estradiol levels followed by a sharp decline and a subsequent rise in progesterone during metestrus and diestrus; however, since estrous stage was determined using vaginal cytology without direct hormonal measurements, inferences about hormonal influences on behavior remain indirect. Research indicates that estradiol administration increases activity levels in both sexes, likely mediated by the ERα pathway (Lightfoot, 2008). Further supporting this link, anxiety-like behaviors have been shown to fluctuate across the estrous cycle, with metestrus and diestrus often associated with higher anxiety-like behavior compared to proestrus, where an inhibition of exploratory activity is observed (Bailey and Crawley, 2009; Pestana and Graham, 2024.)

Interphase differences were observed in habituation-like activities, with distinct patterns of engagement across the estrous cycle during both light and dark cycle. The analysis demonstrates that during the light cycle, females in estrus consistently displayed higher habituation-like activity compared to those in proestrus and diestrus. Though, during the dark cycle, the engagement in this habituation activities were elevated in diestrus instead, compared to estrus (Figures 3F,L, 5K). Previously, it has shown that estrous stage has no influence on female mouse self-grooming behaviors in C57BL/6J male and female mice (Zeng et al., 2023). However, our findings suggest both sex-based and interphase differences, with patterns suggesting that the phases of the estrous cycle influence the propensity for repetitive and familiar behaviors. The increased habituation behaviors observed during estrus during the light cycle may reflect a phase-specific shift in behavioral priorities, potentially linked to heightened environmental responsiveness.

Sleep-related behaviors varied across the estrous phases, with significant differences observed during the light cycle. Females in metestrus exhibited higher engagement in sleep-related activities compared to those in proestrus (Figure 5K). Previous literature suggest that estrogen is a major gonadal hormone influencing sleep/wakefulness, and the proestrus is known to be a phase where estrogen steadily increases, the reduction in sleep-related behavior observed in proestrus may be to that, while in metestrus, a combination of estradiol and progesterone facilitates the recovery of REM sleep after sleep loss (Choi et al., 2021; Caufriez et al., 2011).

While several clusters and factors revealed significant differences between sexes and across estrous phases, many other clusters and activities showed no significant differences. This lack of distinction is not entirely unexpected, given that the mice, regardless of sex or hormonal state, were housed in the same controlled environment with limited external stimuli. Mice are highly adaptive animals, and their behaviors are shaped by intrinsic and environmental factors (Mo et al., 2016). In a stable environment, it is natural for many behaviors to remain consistent across sexes and hormonal phases (Meziane et al., 2007; Zhao et al., 2018; Levy et al., 2023). Additionally, as nocturnal animals, much of their activity rhythm may be regulated by circadian cues rather than solely by sex or hormone levels (Refinetti, 2015; Steel et al., 2024). These non-significant results underscore that while sex and hormonal fluctuations can influence specific behaviors, the fundamental behavioral repertoire of mice remains stable, driven by the shared environmental conditions and their innate nocturnal patterns of activity (Refinetti, 2015; Steel et al., 2024).

The interplay between hormones and behavior in the brain involves many pathways, neural circuitry, and organs to maintain a healthy functional system (Jennings and de, 2020). Both estrogen and progesterone fluctuate together and have various functions throughout the body. These can range from regulating the development and maintenance of reproductive tissues and cardiovascular and neurological systems to enhancing memory, having anxiolytic effects, regulating sleep patterns, etc. The literature on sex differences in activity levels and anxiety behaviors is mixed. For example, Pestana et al. (2022) reported that anxiety-related behaviors, such as increased vigilance, elevated heart rate, inhibition of exploration, etc., were more pronounced in the metestrus and diestrus phases compared to proestrus. Additionally, some studies have shown that female rodents are more active and exploratory in open-field tests, exhibiting greater ambulatory and rearing behaviors, while appearing less anxious than males. However, other findings suggest no difference between sexes in anxiety or activity levels, with some strains of mice showing no sex differences in open-field tasks (Lovick and Zangrossi, 2021). These inconsistencies complicate the interpretation of sex- and cycle-specific activity levels. However, our findings support the idea that during metestrus and diestrus phases, females may engage in more active behaviors. At the same time, males exhibit a more energy-conserving strategy during the dark cycle, which aligns with other reports of strain and sex differences in behavior across various tasks.

### Limitations

One of the limitations of our study is that our facility uses a 14:10 light–dark cycle, which deviates from the standard 12:12 light–dark cycle. Moreover, various factors can influence the reproducibility of research findings, including differences in animal models, strain, genetic background, age, sex, coat color, and environmental conditions. These variables must be carefully considered in future studies to ensure robust results. Another limitation was the relatively short recording period, as long-term isolation can impact behavior (Gris et al., 2017). Extended isolation may affect outcomes, and future research should aim to balance continuous monitoring with the potential effects of prolonged isolation on the animals. Finally, determining the exact stage of the estrous cycle remains challenging due to the continuous changes in vaginal cell types and the absence of clear demarcations between phases (Byers et al., 2012). This introduces a potential risk of misclassification, which could affect the accuracy of the data. Moreover, while proestrus and estrus are typically associated with higher estradiol and metestrus and diestrus are associated with lower estradiol levels (Zeng et al., 2023), we did not directly measure hormonal levels in this study.

## Conclusion

In conclusion, our findings highlight distinct sex- and phase-dependent behavioral differences in C57BL/6J mice, with females showing higher locomotor activity, particularly during estrus, while males engaged more in sleep-related behaviors. Feeding and habituation behaviors were greater in males, with notable inter-estrous phase variations among females. This study adds valuable insights into the link between behavioral differences that stem from sex, reproductive cycle, and circadian rhythm. By utilizing advanced software for behavioral analysis alongside a controlled approach that minimizes bias, our work establishes a reliable and accurate baseline for future research. Moreover, this method’s sensitivity enables the tracking of health changes in mouse models, reinforcing their use as a model for human disease. Moreover, our work helps to bring more female-based animal models into behavioral research.

## Data Availability

The raw data supporting the conclusions of this article will be made available by the authors, without undue reservation.
