# Supplementary material for: Real-time behavioral monitoring of C57BL/6J mice during reproductive cycle
Source: Front Neurosci. 2025 Mar 3;19:1509822. doi: 10.3389/fnins.2025.1509822 (PMC11911481; doi:10.3389/fnins.2025.1509822)
Supplement: Supplementary file 1 [file Data_Sheet_1.pdf]

**Table 1. Definition of detected behavioral activities by the software**

| Activity | Definition | Abbreviation |
|---|---|---|
| Awaken | Any movement from sleep that starts and continues without resumption of sleep | Awaken |
| Chew | Any brief period during eating where the mouth detaches from the food | Chew |
| Come down | Any movement of the animal from a fully reared position to a position | Come Down |
| Come down from partially reared | Any movement of the animal from a partially reared up position to a low level | Come Down from PR |
| Come down to partially reared | Any movement of the animal from a fully reared up position to a partially reared up position | Come Down to PR |
| Dig | Any movement with animal's hind limbs inside bedding and resulting in a considerable movement of the bedding | Dig |
| Drink | In a reared up position. animal's nose/mouth crosses water calibrated line | Drink |
| Eat | In a reared up position, animal's nose/mouth crosses feeding box calibrated line | Eat |
| Forage | Any movement with animal's forepaws and/or mouth inside bedding and resulting in a considerable movement of the bedding, typically in front of the animal | Forage |
| Groom | Deformation of body over a defined criteria and longer than specified time | Groom |
| Hang cuddled | Any movement of the animal resulting in animal having all four limbs at the top of the cage (more horizontal position at the top of the cage) | Hang Cuddled |
| Hang vertically from hang cuddled | Any movement from a hang cuddled position to a hang vertical position | Hang Ver from Hanged Cuddled |
| Hang vertically from rear up | Any movement of the animal resulting in animal leaving the floor and not coming back<br>down immediately and remaining vertical after leaving the floor | Hang Ver From RearUp |
| Jump | Any movement from a lower to a higher position and back to the position | Jump |
| Land vertically | Any movement of the animal from a hanging position with feet off of the floor to the feet coming back down on to the floor | Land Ver |
| Pause | Implemented similar to sleep with those sleep constraints, but lasting only for much smaller prescribed minimum time | Pause |
| Rear up | Any movement of the animal from a low position to a full reared up position | RearUp |
| Rear up from partially reared | Any movement of the animal from a partially reared position to a fully reared up position | ReaurUp from PR |
| Rear up partially | Any movement of the animal from position to a partially reared position | RearUp Part |
| Remain hang cuddled | After hanging cuddled, remain in a hanging cuddled position | Remain Hang Cuddled |
| Remain hang vertically | After hanging vertically remain in a vertical position | Remain Hang Ver |
| Remain partially reared | Remain in a partially reared position | Remain PR |
| Remain reared up | After rearing up, remain in the reared up position | Remain RearUp |
| Repetitive jumping | Any series of successive jump behaviors | Repetitive jump |
| Sleep | The onset of sleep is detected at each instance there is no significant movement for a prescribed amount of time | Sleep |
| Sniff | When the animal is either fully or partially reared, and the tip of the mouth makes some random movements (back and forth, up/down, protrude/retract) | Sniff |
| Stationary | Any sequence for which there is no translational movement | Stationary |
| Stretch body | Any movement from shorter to longer/elongated body (horizontally or vertically) | Stretch |
| Turn | Any movement of the animal from a side view to a front vice versa | Turn |
| Twitch | Any brief movement of the animal during sleep | Twitch |
| Walk left | Any movement of the animal in left direction over a given distance | Walk L |
| Walk right | Any movement of the animal in right direction over a given distance | Walk R |
| Walk slowly | Any sideways movement of animal without a definite direction component | Walk S |

**Table 1.** Retrieved with permission from Yamamoto, K., Gris, K. V., Sotelo Fonseca, J. E., Gharagozloo, M., Mahmoud, S., Simard, C., Cloutier, T., Gris, P., & Gris, D. (2018). Exhaustive Multi-Parametric Assessment of the Behavioral Array of Daily Activities of Mice Using Cluster and Factor Analysis. Frontiers in Behavioral Neuroscience, 12, 348015. https://doi.org/10.3389/fnbeh.2018.00187
