# Supplementary material for: Real-time behavioral monitoring of C57BL/6J mice during reproductive cycle
Source: Front Neurosci. 2025 Mar 3;19:1509822. doi: 10.3389/fnins.2025.1509822 (PMC11911481; doi:10.3389/fnins.2025.1509822)
Supplement: Supplementary file 2 [file Data_Sheet_2.pdf]

Table 2. Significant Differences in Activity across groups across time periods

Activity	Comparison	F-value	p-value	t-stat	Time Period
CD_Frm_Part_Rear	ANOVA	3.684038	0.006952		
CD_Frm_Part_Rear	M vs N		0.025297	-2.27607	24hr
CD_Frm_Part_Rear	D vs N		0.001428	-3.30847	24hr
CD_Frm_Part_Rear	ANOVA	3.624698	0.007643		
CD_Frm_Part_Rear	D vs N		0.001208	-3.36174	24hr
CD_Frm_Part_Rear	P vs N		0.025347	-2.28647	24hr
Dig	ANOVA	8.267541	5.14E-06		
Dig	M vs N		1.50E-05	4.586358	24hr
Dig	D vs N		2.96E-06	5.04352	24hr
Dig	P vs N		0.021341	2.356398	24hr
Dig	E vs N		1.95E-05	4.582956	24hr
Eat	ANOVA	3.420904	0.010576		
Eat	E vs N		0.005967	-2.83608	24hr
Hang_Cuddled	ANOVA	7.334866	2.16E-05		
Hang_Cuddled	M vs P		0.049936	-2.01232	24hr
Hang_Cuddled	M vs E		0.005341	-2.91558	24hr
Hang_Cuddled	M vs N		0.02384	2.300027	24hr
Hang_Cuddled	D vs N		0.003467	3.016248	24hr
Hang_Cuddled	P vs N		0.000167	3.9859	24hr
Hang_Cuddled	E vs N		2.22E-06	5.157758	24hr
Hang_Vert_Frm_HC	ANOVA	7.194841	2.68E-05		
Hang_Vert_Frm_HC	M vs E		0.037272	-2.14103	24hr
Hang_Vert_Frm_HC	M vs N		0.001914	3.200684	24hr
Hang_Vert_Frm_HC	D vs N		0.000319	3.769461	24hr
Hang_Vert_Frm_HC	P vs N		0.000769	3.522576	24hr
Hang_Vert_Frm_HC	E vs N		1.46E-06	5.266865	24hr
Hang_Vert_Frm_Rear_Up	ANOVA	3.143428	0.01644		
Hang_Vert_Frm_Rear_Up	D vs N		0.041526	2.072911	24hr
Hang_Vert_Frm_Rear_Up	E vs N		0.00093	3.457931	24hr
Hang_Vertically	ANOVA	5.260978	0.000561		
Hang_Vertically	M vs N		0.004683	2.902975	24hr
Hang_Vertically	D vs N		0.004468	2.929237	24hr
Hang_Vertically	P vs N		0.023374	2.319572	24hr
Hang_Vertically	E vs N		0.000697	3.548317	24hr
Rear_up_Partially	ANOVA	9.824409	4.92E-07		
Rear_up_Partially	M vs N		7.27E-05	-4.1664	24hr
Rear_up_Partially	D vs N		0.000141	-4.00668	24hr
Rear_up_Partially	P vs N		0.004234	-2.95984	24hr
Rear_up_Partially	E vs N		0.001986	-3.21315	

Remain_Hang_Cuddled	ANOVA	11.09738	7.57E-08		
Remain_Hang_Cuddled	M vs N		3.20E-05	4.387555	24hr
Remain_Hang_Cuddled	D vs E		0.030812	-2.24073	24hr
Remain_Hang_Cuddled	D vs N		1.67E-05	4.594642	24hr
Remain_Hang_Cuddled	P vs N		1.39E-05	4.684994	24hr
Remain_Hang_Cuddled	E vs N		3.22E-09	6.773829	24hr
Remain_Hang_Vert	ANOVA	2.899535	0.024184		
Remain_Hang_Vert	M vs N		0.007195	2.752627	24hr
Remain_Hang_Vert	D vs N		0.005514	2.855908	24hr
Remain_Hang_Vert	P vs N		2.69E-05	4.503949	24hr
Remain_Hang_Vert	E vs N		2.27E-05	4.541606	24hr
Sniff	ANOVA	5.726715	0.000268		
Sniff	M vs N		0.011572	-2.57954	24hr
Sniff	D vs N		0.001675	-3.25708	24hr
Sniff	P vs N		0.014929	-2.49759	24hr
Sniff	E vs N		0.007296	-2.76377	24hr
Walk_Left	ANOVA	10.46418	1.91E-07		
Walk_Left	M vs E		0.011078	-2.64037	24hr
Walk_Left	M vs N		0.00136	3.309698	24hr
Walk_Left	D vs N		2.26E-05	4.513311	24hr
Walk_Left	P vs N		2.83E-05	4.489543	24hr
Walk_Left	E vs N		3.64E-08	6.187608	24hr
Walk_Right	ANOVA	11.00485	8.66E-08		
Walk_Right	M vs E		0.008623	-2.73663	24hr
Walk_Right	M vs N		0.003083	3.044475	24hr
Walk_Right	D vs N		3.11E-06	5.031594	24hr
Walk_Right	P vs N		4.70E-06	4.974678	24hr
Walk_Right	E vs N		3.75E-08	6.180361	24hr
Chew	Daytime	3.049473	0.01908		
Chew	M vs N		0.00753	2.736357	Daytime
Chew	D vs N		0.024088	2.301169	Daytime
Chew	P vs N		0.026721	2.264726	Daytime
Come_Down	Daytime	4.823133	0.001127		
Come_Down	M vs E		0.007836	-2.77291	Daytime
Come_Down	D vs N		0.004466	2.929428	Daytime
Come_Down	P vs N		0.026061	2.275044	Daytime
Come_Down	E vs N		0.000365	3.746169	Daytime
CD _Frm_Part_Rear	Daytime	3.413764	0.010697		
CD _Frm_Part_Rear	M vs N		0.027736	-2.23858	Daytime
CD _Frm_Part_Rear	D vs E		0.013249	-2.59538	Daytime
CD _Frm_Part_Rear	D vs N		0.002663	-3.10499	Daytime
Dig	Daytime	3.413305	0.010705		
Dig	M vs N		0.017577	2.420529	Daytime

Dig	D vs N		0.000926	3.445639	Daytime
Dig	P vs N		0.005698	2.855188	Daytime
Dig	E vs N		1.63E-05	4.631378	Daytime
Groom	Daytime	2.759293	0.030166		
Groom	D vs N		0.005781	2.839229	Daytime
Groom	P vs N		0.020888	2.36503	Daytime
Hang_Cuddled	Daytime	4.060595	0.003819		
Hang_Cuddled	D vs N		0.013154	2.538394	Daytime
Hang_Cuddled	E vs N		0.000349	3.760139	Daytime
Hang_Vert_Frm_HC	Daytime	3.397932	0.011004		
Hang_Vert_Frm_HC	D vs N		0.00355	3.008126	Daytime
Hang_Vert_Frm_HC	E vs N		5.07E-05	4.319033	Daytime
HangVert_Frm_Rear_Up	Daytime	2.853529	0.026005		
HangVert_Frm_Rear_Up	M vs E		0.014473	-2.5354	Daytime
HangVert_Frm_Rear_Up	D vs N		0.03536	2.14194	Daytime
HangVert_Frm_Rear_Up	E vs N		0.007481	2.754711	Daytime
HangVertically	Daytime	3.351811	0.011806		
HangVertically	D vs N		0.016751	2.445438	Daytime
HangVertically	E vs N		0.001642	3.275444	Daytime
Jump	Daytime	3.749435	0.006263		
Jump	M vs N		0.038295	2.103662	Daytime
Jump	D vs N		0.000214	3.886369	Daytime
Jump	P vs N		0.011517	2.596995	Daytime
Jump	E vs N		0.00106	3.416641	Daytime
Rear_Up	Daytime	2.576708	0.040168		
Rear_Up	M vs E		0.005307	-2.91789	Daytime
Rear_Up	E vs N		0.011425	2.597941	Daytime
Rear_up_Partially	Daytime	8.833842	2.17E-06		
Rear_up_Partially	M vs N		0.000242	-3.82864	Daytime
Rear_up_Partially	D vs N		0.000214	-3.88706	Daytime
Rear_up_Partially	P vs N		0.002615	-3.12491	Daytime
Rear_up_Partially	E vs N		0.011422	-2.59805	Daytime
Sniff	Daytime	3.889712	0.005006		
Sniff	M vs N		0.037328	-2.11457	Daytime
Sniff	D vs E		0.00603	-2.90448	Daytime
Sniff	D vs N		0.000875	-3.46303	Daytime
Sniff	P vs N		0.033005	-2.17636	Daytime
Walk_Left	Daytime	3.99329	0.004243		
Walk_Left	M vs E		0.046396	-2.04353	Daytime
Walk_Left	D vs N		0.006565	2.794084	Daytime
Walk_Left	P vs N		0.016132	2.467419	Daytime
Walk_Left	E vs N		0.001247	3.364595	Daytime
Walk_Right	Daytime	4.565325	0.001701		

Walk_Right	M vs E		0.041364	-2.09499	Daytime
Walk_Right	D vs N		0.002066	3.188867	Daytime
Walk_Right	P vs N		0.003207	3.055662	Daytime
Walk_Right	E vs N		0.001569	3.290388	Daytime
CD_To_Part_Rear	Nighttime	3.231063	0.014304		
CD_To_Part_Rear	D vs N		0.000954	-3.4362	Nighttime
CD_To_Part_Rear	P vs N		0.038791	-2.1072	Nighttime
Dig	Nighttime	6.862049	4.50E-05		
Dig	M vs N		6.93E-05	4.179502	Nighttime
Dig	D vs N		8.77E-05	4.141278	Nighttime
Dig	E vs N		0.011687	2.589383	Nighttime
Eat	Nighttime	4.161959	0.00324		
Eat	M vs N		0.026111	-2.26322	Nighttime
Eat	D vs N		0.018901	-2.39812	Nighttime
Eat	E vs N		0.009827	-2.65439	Nighttime
Groom	Nighttime	3.829417	0.005512		
Groom	M vs N		0.001425	-3.29509	Nighttime
Groom	P vs N		0.011833	-2.58673	Nighttime
Groom	E vs N		0.006208	-2.82194	Nighttime
Hang_Cuddled	Nighttime	6.572858	7.07E-05		
Hang_Cuddled	M vs E		0.006829	-2.82455	Nighttime
Hang_Cuddled	M vs N		0.044258	2.041226	Nighttime
Hang_Cuddled	D vs N		0.010081	2.638221	Nighttime
Hang_Cuddled	P vs N		0.000814	3.504709	Nighttime
Hang_Cuddled	E vs N		1.93E-05	4.584928	Nighttime
Hang_Vert_Frm_HC	Nighttime	6.417927	9.02E-05		
Hang_Vert_Frm_HC	M vs N		0.001801	3.22044	Nighttime
Hang_Vert_Frm_HC	D vs N		0.001233	3.355241	Nighttime
Hang_Vert_Frm_HC	P vs N		0.002435	3.148874	Nighttime
Hang_Vert_Frm_HC	E vs N		2.60E-05	4.504026	Nighttime
Hang_Vert_Frm_Rear_Up	Nighttime	2.613848	0.0379		
Hang_Vert_Frm_Rear_Up	M vs N		0.036323	2.126164	Nighttime
Hang_Vert_Frm_Rear_Up	E vs N		0.002034	3.20527	Nighttime
Hang_Vertically	Nighttime	4.402175	0.002207		
Hang_Vertically	M vs N		0.003308	3.02098	Nighttime
Hang_Vertically	D vs N		0.019013	2.395782	Nighttime
Hang_Vertically	P vs N		0.031403	2.197384	Nighttime
Hang_Vertically	E vs N		0.002085	3.197083	Nighttime
Pause	Nighttime	7.189548	2.70E-05		
Pause	M vs N		0.00018	-3.91412	Nighttime
Pause	P vs N		0.002859	-3.09468	Nighttime
Pause	E vs N		0.001707	-3.26287	Nighttime
Rear_up_Partially	Nighttime	4.971167	0.00089		

Rear_up_Partially	M vs N		0.002066	-3.17601	Nighttime
Rear_up_Partially	D vs N		0.01034	-2.62881	Nighttime
Rear_up_Partially	E vs N		0.01204	-2.57811	Nighttime
Remain_Hang_Cuddled	Nighttime	11.28322	5.78E-08		
Remain_Hang_Cuddled	M vs E		0.043954	-2.06786	Nighttime
Remain_Hang_Cuddled	M vs N		1.68E-05	4.55781	Nighttime
Remain_Hang_Cuddled	D vs E		0.025446	-2.32369	Nighttime
Remain_Hang_Cuddled	D vs N		1.61E-05	4.604231	Nighttime
Remain_Hang_Cuddled	P vs N		1.82E-05	4.610632	Nighttime
Remain_Hang_Cuddled	E vs N		1.38E-08	6.4233	Nighttime
Remain_Hang_Vert	Nighttime	3.228069	0.014373		
Remain_Hang_Vert	M vs N		0.003446	3.007295	Nighttime
Remain_Hang_Vert	D vs N		0.004488	2.927684	Nighttime
Remain_Hang_Vert	P vs N		4.81E-06	4.968594	Nighttime
Remain_Hang_Vert	E vs N		4.77E-06	4.958782	Nighttime
Sniff	Nighttime	4.899537	0.000998		
Sniff	M vs N		0.022608	-2.32131	Nighttime
Sniff	D vs N		0.006902	-2.77619	Nighttime
Sniff	P vs N		0.040109	-2.09271	Nighttime
Sniff	E vs N		0.003569	-3.01583	Nighttime
Stretch_Body	Nighttime	6.2619	0.000115		
Stretch_Body	M vs N		1.64E-05	4.564175	Nighttime
Stretch_Body	D vs N		0.001511	3.290278	Nighttime
Stretch_Body	P vs N		0.000429	3.703076	Nighttime
Stretch_Body	E vs N		0.006804	2.789018	Nighttime
Walk_Left	Nighttime	10.75164	1.25E-07		
Walk_Left	M vs E		0.026382	-2.28973	Nighttime
Walk_Left	M vs N		8.19E-05	4.133884	Nighttime
Walk_Left	D vs E		0.040018	-2.12454	Nighttime
Walk_Left	D vs N		1.25E-05	4.670748	Nighttime
Walk_Left	P vs N		1.12E-06	5.348143	Nighttime
Walk_Left	E vs N		5.58E-08	6.083366	Nighttime
Walk_Right	Nighttime	12.02941	1.97E-08		
Walk_Right	M vs E		0.012043	-2.60786	Nighttime
Walk_Right	M vs N		0.000131	4.003431	Nighttime
Walk_Right	D vs E		0.026687	-2.30319	Nighttime
Walk_Right	D vs N		6.73E-06	4.832659	Nighttime
Walk_Right	P vs N		2.58E-07	5.722463	Nighttime
Walk_Right	E vs N		9.92E-09	6.503258	Nighttime
